# Surveillance for ototoxicity in platinum-based chemotherapy using mobile health audiometry with extended high frequencies

**DOI:** 10.1017/S0022215122001281

**Published:** 2023-01

**Authors:** K Ehlert, B Heinze, M A Graham, D Swanepoel

**Affiliations:** 1 Department of Speech-Language Pathology and Audiology, Sefako Makgatho Health Sciences University, Pretoria, South Africa; 2 Department of Speech-Language Pathology and Audiology, Mathematics and Technology Education, University of Pretoria, Pretoria, South Africa; 3 Department of Science, Mathematics and Technology Education, University of Pretoria, Pretoria, South Africa; 4 Ear Science Institute Australia, Subiaco, Australia

**Keywords:** Chemotherapy Agent, Cancer, Carboplatin, Cisplatin, Oxaliplatin, Hearing Disorder, Ototoxicity, Drug Induced

## Abstract

**Objective:**

This study investigated mobile health enabled surveillance in ototoxicity.

**Method:**

This was a longitudinal study of 32 participants receiving chemotherapy. Baseline and exit audiograms that included conventional and extended high frequency audiometry were recorded within the patient's treatment venue using a validated mobile health audiometer.

**Results:**

Average hearing thresholds at baseline were within the normal range (81.2 per cent left; 93.8 per cent right), reducing at exit testing (71.9 per cent left; 78.1 per cent right). Half of participants presented with a threshold shift according to ototoxicity monitoring criteria. The frequencies affected the most were between 4000 and 16 000 Hz, with left ears significantly more affected than right ears. Noise levels exceeded the maximum permissible ambient noise levels in up to 43.8 per cent of low frequencies (250–1000 Hz).

**Conclusion:**

Mobile health supported audiometry proved to be an efficacious tool for ototoxicity monitoring at the treatment venue. Changes in hearing ability over time could be tracked, improving surveillance in patients with full treatment schedules.

## Introduction

Cancer is known to be one of the major illnesses in the world, resulting in about 19.3 million new cases and 10 million deaths in 2020. The overall number of individuals living within 5 years of a cancer diagnosis, called the 5-year prevalence, is estimated to be 50.6 million worldwide.^1^ Although cancer appears to be a life-altering diagnosis, there has been an overall decrease of 26 per cent in cancer deaths in the last two decades because of medical advancements.^2^ However, treatment outcomes can also lead survivors to have long-term physical and psychological issues.^3^ For this reason, for those who are transitioning to a life with and beyond cancer, there is a need to assess how these long-term consequences affect their health-related quality of life (QoL).

Ototoxic medications typically used in chemotherapy can result in cochleotoxicity, vestibulotoxicity or both.^4,5^ Ototoxicity refers to any hearing deficit or tinnitus resulting from acute or permanent inner ear dysfunction following treatment with an ototoxic drug. Platinum-based compounds (cisplatin, carboplatin and oxaliplatin) are used as single agents and in combination with other drugs for the treatment of various types of cancer (e.g. testicular carcinoma, lung carcinoma, ovarian carcinoma, head and neck carcinomas, melanomas, lymphomas, and neuroblastomas).^6,7^

The platinum-based drugs combine DNA and result in irreversible changes that prohibit tumour cell division. Common adverse effects of platinum-based drugs include nephrotoxicity and ototoxicity.^8^ When ototoxins cross the blood-labyrinth barrier of the auditory system, the barrier breaks down and instantly causes loss of endolymphatic potential that leads to the demise of auditory hair cells in the cochlea.^4^ Furthermore, genetic mutations that cause mitochondrial pathologies, are often associated with hearing loss, and substances such as cisplatin are known to damage mitochondria,^8^ which results in elevation of sensory thresholds and eventually hearing loss.^4^ Hearing changes are typically detected in the highest audible frequencies progressing to lower frequencies with additional ototoxicity exposure. Consequently, cancer survivors often have difficulties understanding speech in noise.^9^

Unfortunately, ototoxic hearing loss may go unnoticed by patients until a communication problem becomes apparent, suggesting that hearing loss within the frequency range important for speech understanding has already occurred.^10^ For patients with life-threatening illnesses that warrant treatment with ototoxic drugs, communication ability is a central QoL issue. These patients have important communication needs in terms of dealing with multiple healthcare professionals and family members during the course of cancer treatment.^11^ Therefore, identifying ototoxic damage early can improve treatment outcomes by minimising hearing loss progression and its associated impact on functioning in daily life.^4^ Early identification and monitoring of ototoxicity can also provide hearing care professionals with the opportunity to perform appropriate (re)habilitation during and after treatment.^10^

The only way to detect ototoxicity is by assessing auditory function directly.^4^ For patients undergoing chemotherapy, the difficulties of introducing an ototoxicity monitoring protocol include fatigue, general acute illness, travel problems and priority issues.^10^ Present ototoxicity testing recommendations include detailed test protocols conducted in a sound-treated room by an audiologist.^12^ Moving patients who are undergoing chemotherapy into a sound-treated room is usually not feasible because of their immunocompromised state and overburdened treatment schedule.^12^ This contributes to the ineffectiveness of existing monitoring programmes.

Mobile solutions to test hearing on digital devices like smartphones have proven effective for hearing assessment outside of conventional clinic environments and provide a low-cost alternative to conventional ototoxicity monitoring that requires the patient to attend an audiology clinic.^13,14,15^ These mobile health technologies are often also designed to be used by minimally trained persons, which can further improve access to hearing care.^14,16^ Automated pure tone testing protocols using mobile health technologies with calibrated headphones demonstrate clinical hearing threshold assessments (at the conventional frequencies as well as extended high frequencies) comparable with conventional testing with improved efficiency, noise monitoring and quality control.^17^ Smartphone audiometry has also provided reliable results in an infectious disease clinic setting and can be used as a baseline and monitoring tool.^13^ Employing mobile health tools connected to cloud-based data management systems allows for paperless tracking of patient data and potential threshold shifts.^13^ The application allows for remote hearing testing where patient data and results can be uploaded onto centralised cloud-based servers for data management through cellular networks. Patients can also be linked to the closest audiologist for further management.^13^

As cancer patients face unique health problems and side effects throughout the course of platinum-based chemotherapy treatment, a flexible approach to ototoxicity monitoring is required. Hearing testing, particularly within a clinic or hospital setting, is required to overcome patient challenges and to implement a successful ototoxicity monitoring programme. The mobile nature, quality controls, use by healthcare workers and paperless surveillance in the cloud makes mobile health supported devices ideal for ototoxicity surveillance. Hearing testing during chemotherapy treatment within hospital wards and oncology clinics will relieve the already over-burdened treatment schedule of cancer patients. This study therefore investigated platinum-based chemotherapy ototoxicity surveillance using mobile health audiometry.

## Materials and methods

Ethical clearance was obtained from the research ethics committee of the Faculty of Health Sciences and Faculty of Humanities of the University of Pretoria on 11 January 2019 (approval number: 665/2018).

### Study design, setting and participants

A longitudinal study design was implemented. Inclusion criteria were all participants (aged more than 10 years) treated with platinum-based compounds (cisplatin, carboplatin or oxaliplatin) for the first time in private and public oncology units and hospitals. Testing was conducted during chemotherapy treatment in oncology clinics or at the hospital bedside. Thirty-two participants (64 ears) above the age of 10 years (to ensure reliable behavioural testing) participated in the study, taking into account that repeated measures (baseline and exit testing) were performed for each participant.

### Equipment

The ‘ototoxicity monitoring case history interview’^18,19^ was used as a guideline during case history at baseline testing. The Heine Mini 3000 Otoscope (Gilching, Germany) was used to perform otoscopy prior to pure tone testing. The hearTest® certified digital audiometer was used for testing. The hearTest® extended high frequencies application was used on a Samsung A3 smartphone with the Android version 8.0 operating system (Google, Mountain View, USA). Supra-aural Sennheiser HDA 300 headphones (Sennheiser, Wedemark, Germany; K Venter, unpublished thesis) calibrated according to prescribed standards (International Organisation for Standardisation 389–1, 2017)^20^ and adhering to equivalent threshold sound pressure levels determined for this headphone were connected to the smartphone. Daily calibration listening checks of headphones were performed. The hearTest audiometer has been validated to monitor noise accurately using the smartphone microphone (JJ van Tonder, unpublished thesis).

There was real-time monitoring of noise with the smartphone microphone to alert the user of environmental noise concerns during testing. The maximum permissible ambient noise levels used for HDA 300 headphones were 22.7, 19.4, 22.8, 25.1, 38.8 and 36.2 dB for 250, 500, 1000, 2000, 4000 and 8000 Hz, respectively, for testing at the minimum response level of 10 dB HL. Automated pre-programmed test sequences (250–16 000 Hz) were used for improved efficiency, and the reliability of patient responses was monitored throughout (hearX Group, Pretoria, South Africa). Testing commenced and ended at 1000 Hz frequency in each ear. Threshold concern was flagged at 1000 Hz when there was a difference of equal to or more than 10 dB (hearX Group). Patient, test and facility data were consolidated instantly on a secure online database. Data collected by the smartphone were automatically uploaded to a secure cloud-based server once connected to wi-fi. Access to the smartphone and cloud-based data were protected by a user password.

### Data collection procedures

For this study, baseline testing included case history, otoscopy and pure tone audiometry (conventional air conduction and extended high frequency). Exit testing included otoscopy and pure tone audiometry (conventional air conduction and extended high frequency).

Testing was not performed in a sound-treated room but was instead performed in the oncology rooms during chemotherapy appointments or oncology visits as well as in hospital wards. Participants were tested prior to initiation of treatment or within 24-hours of treatment initiation (baseline testing). Post-treatment follow up occurred at three to six months post-treatment (exit testing). Prior to baseline testing, participants were provided with simple instructions and a demonstration regarding the testing procedure. An automated hearTest protocol was employed for baseline and exit testing to determine participant thresholds. The shortened threshold ascending method was used in the automated protocol to obtain thresholds (K Venter, unpublished thesis).

The pure tone average (PTA) was calculated as the better ear average for four frequencies of 500, 1000, 2000 and 4000 Hz. The World Health Organization grades of hearing impairment were used to determine severity of hearing loss. A PTA of less than 25 dB HL indicates normal hearing, 26–40 dB HL indicates slight hearing loss, 41–60 dB HL indicates moderate hearing loss, 61–80 dB HL indicates severe hearing loss and more than 81 dB HL indicates profound hearing loss.^21^

Threshold shifts were regarded as significant if there was a 20 dB decrease or greater at one frequency, 10 dB decrease or greater at two adjacent frequencies and loss of response at three consecutive frequencies where there was a previously recorded response.^22^ Participants with changes in hearing were advised to continue monitoring until hearing had stabilised and up to 12 months post-treatment.^23^ All participants, even those without a significant shift in threshold were advised to continue annual monitoring of hearing abilities.

### Data analysis

Descriptive statistics (averages and standard deviation) were used to determine the decline in hearing thresholds from baseline to exit testing. The Shapiro–Wilk test^24^ was used to test for normality, and because the *p*-values were less than 0.05, the data differed from normality and non-parametric tests were used. The correlation between the most common frequencies affected and duration between baseline and exit testing was determined. Within-subject statistical tests (Wilcoxon signed rank test) was used to determine the statistical significance of the hearing threshold shifts from baseline to exit testing. If the *p*-value was less than 0.05, then there was a statistically significant difference between baseline and exit. Non-parametric Spearman correlations were used to report on statistically significant (*p* < 0.05) correlations. Because males and females were independent groups, the Mann–Whitney test was used to determine whether males or females differed significantly (*p* < 0.05) in terms of incidence of ototoxicity.

## Results

Table 1 describes the characteristics of the participants (*n =* 32). Case history at baseline testing yielded reports of noise exposure, pre-existing hearing loss and tinnitus. Tinnitus was reported by 34.4 per cent (*n =* 11) of participants prior to chemotherapy treatment, and all these participants also reported an increase in tinnitus during the course of treatment. All participants (100 per cent; *n =* 32) reported an awareness of tinnitus during treatment, and 81.3 per cent (*n =* 26) reported tinnitus symptoms at exit testing.

**Table 1.** Characteristics of participants*

Characteristics	Value
Gender (*n* (%))	
– Male	16 (50.0)
– Female	16 (50.0)
Age (mean (SD; IQR))	47 (16.7; 22)
Age (range; years)	11–70
Age group (*n* (%))	
– 11–15 years	3 (9.4)
– 25–29 years	3 (9.4)
– 30–39 years	3 (9.4)
– 41–49 years	7 (21.9)
– 50–59 years	9 (28.1)
– 61–69 years	4 (12.6)
– 70–74 years	3 (9.4)
Type of cancer (*n* (%))	
– Lymphoma	6 (18.8)
– Cervix cancer	5 (15.6)
– Lung cancer	4 (12.5)
– Breast cancer	3 (9.4)
– Gastric cancer	3 (9.4)
– Colon cancer	3 (9.4)
– Oesophagus cancer	2 (6.3)
– Breast and lymph cancer	1 (3.1)
– Bladder cancer	1 (3.1)
– Prostrate cancer	1 (3.1)
– Seminoma	1 (3.1)
– Cholangiocarcinoma	1 (3.1)
– Tongue cancer	1 (3.1)
– Days between baseline and exit testing (mean (SD; IQR); days)	217 (105.8; 200)
Platinum-based chemotherapy compounds (*n* (%))	
– Cisplatin	14 (43.8)
– Carboplatin	14 (43.8)
– Oxaliplatin	9 (28.0)
Combination treatments^†^ (*n* (%))	
– Combination 1 (cisplatin and oxaliplatin)	3 (9.4)
– Combination 2 (cisplatin, carboplatin and oxaliplatin)	1 (3.1)
Mean dosages of platinum-based compounds	
– Cisplatin (mean dose (SD); mg)	507 (194.8)
– Cisplatin dose (range; mg)	200–825
– Carboplatin (mean dose (SD); mg)	212.4 (1325)
– Carboplatin dose (range; mg)	169–4338
– Oxaliplatin (mean dose (SD); mg)	948.2 (438.8)
– Oxaliplatin dose (range; mg)	180–2040
Otoscopic examination (*n* (%))	
– Normal outer and middle ear	29 (90.6)
– Cerumen impaction (treated prior to exit testing)	2 (6.2)
– Perforation	1 (3.1)
Hearing change from baseline to exit testing	
– 20 dB decrease or greater at one frequency	10 (31.3)
– 10 dB decrease or greater at two adjacent frequencies	15 (46.9)
– Loss of response at 3 consecutive frequencies where there was a previously recorded response	1 (3.1)

*Patients *n =* 32; ^†^of 32 patients, 4 had combination treatments. IQR = inter-quartile range; SD = standard deviation

Half of the participants (50 per cent; *n =* 16) presented with a threshold shift according to ototoxicity criteria from baseline to exit testing. Table 2 summarises the outcomes for pure tone audiometry at baseline and exit testing. Noise levels exceeded the maximum permissible ambient noise levels at the lower frequencies (250–1000 Hz). Test–retest checking at 1000 Hz for differences of 10 dB or greater indicated concerns in 17.2 per cent (*n =* 11 by 10 dB) at baseline testing and 10.9 per cent (*n =* 6 by 10 dB; *n =* 1 by 15 dB) at exit testing in either left or right ears. Hearing thresholds demonstrated a decline from baseline to exit testing with a significant difference in PTA from baseline to exit testing in both the left and right ears (*p =* 0.001). Males were more affected than females; however, the differences were statistically insignificant. The mean PTA difference from baseline to exit testing in the left ears was 4.2 dB (standard deviation (SD) = 4.2 dB, interquartile range = 3.7) and 3.6 dB (SD = 4.6 dB, interquartile range = 6.2) in the right ears.

**Table 2.** Description and outcomes of pure tone testing for baseline and exit testing*

Parameter	Details	Baseline testing	Exit testing	Statistical significance from baseline to exit testing
Mean threshold concern at 1000 dB when difference ≥10 dB (%)	– Left ears	18.8	9.4	
	– Right ears	15.6	12.5	
	– Either left or right ears^†^	17.2	10.9	
Frequencies that exceeded MPANLs^‡^ (%)	Left and right ears			
	– 250 Hz	40.6	43.8	
	– 500 Hz	26.6	28.1	
	– 1000 Hz	39.1	0.0	
	– 10 000 Hz	1.6	1.6	
	– 12 500 Hz	1.6	0.0	
Mean levels by which the MPANLs exceeded the thresholds	Left ears (mean (SD))			
	– 250 Hz	6.8 (4.2)	7.4 (4.3)	
	– 500 Hz	6.0 (5.7)	4.5 (2.6)	
	– 1000 Hz	3.8 (1.9)	3.1 (2.3)	
	– 10 000 Hz	9.0 (3.0)	6.0 (2.4)	
	– 12 500 Hz	3.0 (1.7)	0.0 (0.0)	
	Right ears (mean (SD))			
	– 250 Hz	6.3 (3.6)	7.8 (5.2)	
	– 500 Hz	4.6 (2.6)	5.7 (7.5)	
	– 1000 Hz	5.5 (3.6)	4.0 (4.3)	
PTA (mean (SD; IQR)	Left ears	17.8 (7.8; 10.8)	21.5 (6.9; 11.0)	0.001**
	Right ears	18.5 (11.1; 7.3)	22.1 (12.4; 9.6)	
Degrees of hearing loss (%)	Left ears			
	– Normal	81.2	71.9	
	– Mild	18.8	28.1	
	Right ears			
	– Normal	93.8	78.7	
	– Mild	3.1	18.8	
	– Severe	3.1	3.1	

*Patients *n =* 32; ^†^*n* = 64; ^‡^*n* = 64; **Statistically significant difference from baseline to exit testing. The average of 500, 1000, 2000 and 4000 Hz was used to calculate the pure tone average (PTA). A *p*-value < 0.05 was used to indicate if there is a statistically significant difference between baseline and exit testing. MPANLs = maximum permissible ambient noise levels; SD = standard deviation; IQR = interquartile range

**Fig. 1.**
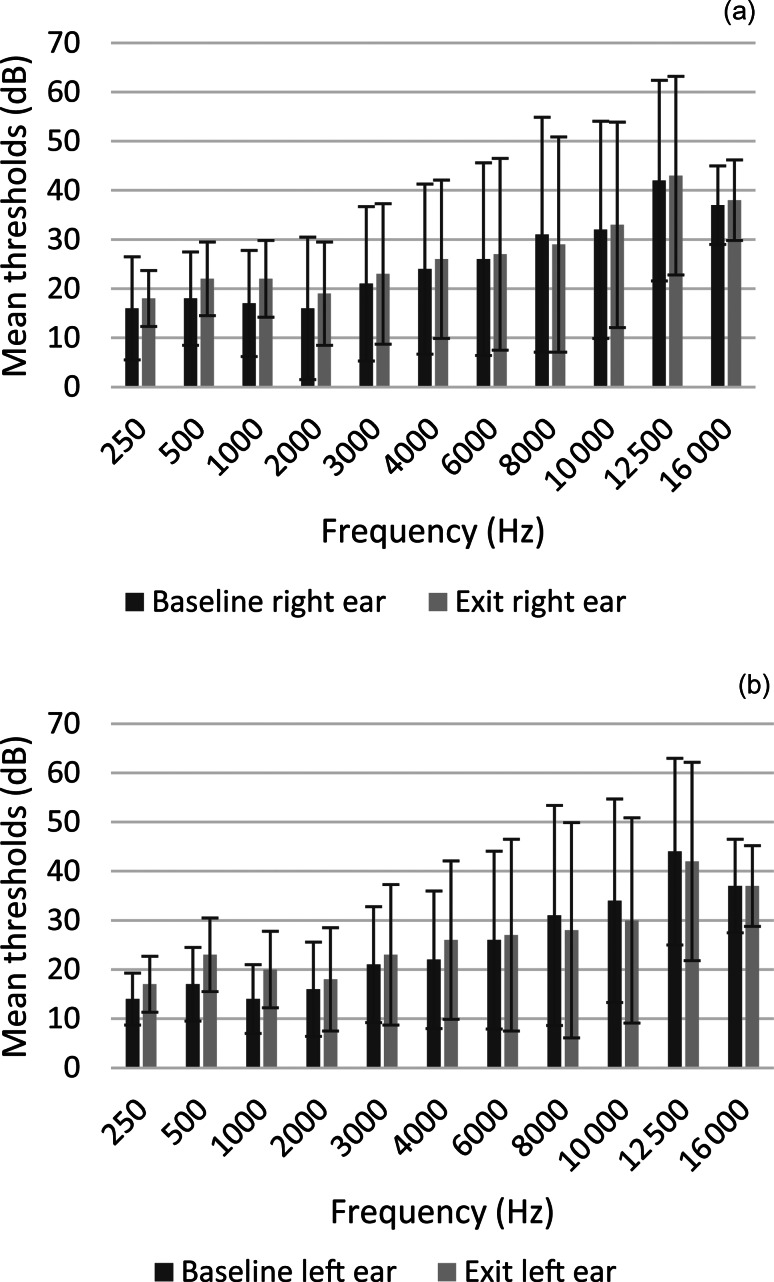
Mean frequency-specific thresholds for baseline and exit testing and error bars showing difference between baseline and exit testing.

Figure 1 illustrates the mean thresholds per frequency for baseline and exit audiometry. Significant deterioration was observed at 250 Hz (*p =* 0.003), 500 Hz (*p =* 0.001), 1000 Hz (*p <* 0.001), 2000 Hz (*p =* 0.024), and 4000 Hz (*p =* 0.011) in left ears and 500 Hz (*p =* 0.031) and 1000 Hz (*p =* 0.001) in right ears from baseline to exit testing. Although not always showing a significant shift, the most affected frequencies according to ototoxicity monitoring criteria were in the high frequencies from 4000 to 16 000 Hz, emphasising the importance of including extended high frequencies in ototoxicity surveillance protocols.

Table 3 demonstrates the most substantial shifts from baseline to exit testing in cisplatin and carboplatin treatment cases.

**Table 3.** Mean PTA differences from baseline to exit testing for specific platinum-based compounds

Treatment	Baseline testing	Exit testing
Carboplatin*: PTA (mean (SD, IQR; dB)		
– Left ears	18.5 (8.7; 15.5)	24.0 (7.3; 11.5)
– Right ears	21.6 (16.4; 11.3)	27.2 (16.8; 7.4)
Cisplatin^†^: PTA (mean (SD, IQR; dB)		
– Left ears	16.0 (6.9; 3.98)	18.5 (2.99; 5.9)
– Right ears	15.6 (3.97; 5.3)	17.95 (4.7; 7.4)
Oxaliplatin^‡^: PTA (mean (SD, IQR; dB)		
– Left ears	18.2 (9.7; 16.3)	23.3 (9.8; 15.0)
– Right ears	18.9 (5.8; 9.6)	19.7 (7.2; 11.7)

*n = 13; ^†^*n* = 10; ^‡^*n* = 5. Participants (*n* = 4) on combined treatments were excluded. PTA = pure tone average; SD = standard deviation; IQR = interquartile range

## Discussion

As long as the best evidence-based practice for the treatment of certain cancers includes treatment with platinum-based compounds, ototoxic hearing loss will need to be considered as a likely side-effect.^4,25^ For cancer patients, hearing monitoring should be performed at the patient's treatment venue.^26^ The mobile health-supported device used in the current study has proved successfully to provide ototoxicity monitoring at the patient's treatment venue. Mobile audiometry applications with automated test sequences, integrated noise monitoring, data capturing and data sharing makes asynchronous ototoxicity monitoring possible and can be facilitated onsite by minimally trained persons.^13^ This could minimise the already full treatment schedule of cancer patients as monitoring can take place during in- or out-patient chemotherapy treatments. This may also address the issue of loss to follow up, as the prolonged effect of chemotherapy on hearing requires long-term monitoring.

Half (50.0 per cent) of the participants in the current study presented with a significant hearing threshold shift from baseline to exit testing. Studies have reported that on average 60–70 per cent of adults treated with cisplatin have ototoxicity,^27^ 20 per cent of patients treated with carboplatin have ototoxicity and ototoxicity from oxaliplatin is typically rare.^23^ Using a mobile health audiometry application supported the ototoxicity monitoring conducted at baseline and exit testing within multiple oncology units and hospital wards. Hearing testing was possible without cancer patients being required to attend audiology clinics.

Extended high frequency testing was included for surveillance purposes using the mobile health audiometry application. The current study found that 4000–16 000 Hz showed the largest average threshold shifts from baseline to exit testing according to ototoxicity monitoring criteria. Extended high frequency testing allows for early identification of hearing disorders before changes are seen in conventional pure tone audiometry and, subsequently, before speech understanding is compromised.^28^

The extended high frequency mobile health audiometry used in this study tested up to 16 000 Hz at a maximum output of 40–60 dB.^23,26,29^ A study by Singh *et al*.^29^ demonstrated that hearing loss was much more common in patients receiving potentially ototoxic drugs (gentamicin, amikacin or cisplatin), in the 10 000–20 000 Hz range (70.1 per cent) than in the 250–8000 Hz range (29.9 per cent). Extended high frequency hearing loss prevalence from baseline to exit testing was 71.4 per cent for cisplatin cases compared with 28.6 per cent in the conventional test frequency range. Consequently, patients in whom hearing is successfully monitored for ototoxicity with extended high frequency are those with better baseline hearing (i.e. responses within the normal range at extended high frequency) and greater post-exposure hearing changes. Although statistically significant (*p <* 0.05), changes from baseline to exit testing were not evident in this study for extended high frequencies, threshold shifts up to 4.9 dB according to ototoxicity threshold shift criteria were evident. This may be because of extended high frequency thresholds that were affected (threshold at maximum extended high frequency intensity for the device) at baseline testing for 59.0 per cent of ears tested in this study. Singh *et al*.^29^ found that most of the patients in the oldest group (51 to 70 years) showed no response on extended high frequency testing, both before and after drug exposure, because of presbycusis. Half (50.0 per cent) of participants in this study were in the oldest age group above 50 years of age and showing high frequency hearing loss at baseline testing. This highlights a limitation of extended high frequency testing with thresholds often absent, especially in older persons, which make it unavailable for monitoring purposes.

Frequencies significantly affected were 250, 500, 1000, 2000 and 4000 Hz in left ears and 500 and 1000 Hz in right ears from baseline to exit testing. The low frequencies in this study also showed that there was a significant difference from baseline to exit testing. Noise levels also affected the lower frequencies which could have resulted in the significant differences from baseline to exit testing. A significant average deterioration of PTA from baseline to exit was evident in this study across left and right ears. The left ear frequency-specific deterioration was more significantly affected compared with the right ears. A study examining the role of extended high frequency testing in ototoxicity monitoring among the 45 patients affected by ototoxicity also observed hearing loss was unilateral (31.1 per cent; *n =* 14) before bilateral hearing loss was reported.^29^ Observing shifts in one ear provides the opportunity to adjust the patient's drug regimen to prevent or limit progression to the other ear.

Most participants in the current study had normal hearing (according to conventional PTA) at baseline testing, and degrees of hearing remained the same at exit testing. The frequencies showing the largest average threshold shift in this study were 2000, 3000, 4000 and 6000 Hz, although they were not significantly different from baseline to exit testing. Platinum-induced hearing loss is reported as initially affecting higher frequencies (equal to or more than 4000 Hz).^23^ Therefore, a shift in hearing threshold is not always evident using the conventional PTA (average of 5000, 1000, 2000 and 4000 Hz). Consequently, mobile health supported devices should include calculations of high frequency PTA (average of 2000, 4000 and 6000 Hz) and potentially extended high frequency PTA (average of 10 000, 12 000, 14 000 and 16 000 Hz) in cases where baseline extended high frequency thresholds could be obtained.^30^

- As long as treatment of certain cancers includes treatment with platinum-based compounds, ototoxic hearing loss will need to be considered as a likely side-effect
- Ototoxicity monitoring for chemotherapy patients is challenging in traditional settings and could be supported by mobile and automated technologies
- This study investigated mobile health enabled surveillance in ototoxicity
- This study showed the usefulness of mobile health conventional and extended high frequency audiometry in ototoxicity surveillance
- Changes in hearing ability over time could be tracked by employing baseline and exit testing
- Monitoring can take place at the treatment venue and decrease the patient's already over-burdened treatment schedule

The mobile health audiometry application monitored environmental noise during threshold testing because testing was performed outside a sound-treated environment.^13^ Frequencies that exceeded maximum permissible ambient noise levels were in the lower frequencies in this study. This may be attributed to testing outside a sound-treated room and the effect of environmental noise. Noise concerns were predominantly noted in this study at 250, 500 and 1000 Hz. The mean levels by which the maximum permissible ambient noise levels exceeded the thresholds was 3.0–7.8 dB, which emphasises that maximum permissible ambient noise levels were exceeded by a small margin on average. Considering the convenience, and often the only option, for ototoxicity surveillance to take place is at the cancer patient's treatment venue, the possible noise interference at the lower frequencies highlights the need to focus on the high frequencies to detect threshold shifts in these settings. As the most sensitive frequencies for ototoxicity are in the high frequencies,^4^ it could mitigate concerns of noise levels affecting the results when testing during chemotherapy in- and out-patient appointments as an early detection measure. Longer duration of platinum-based treatment also eventually affects the mid and lower frequencies so this should be kept in mind.^4,25^ It also highlights the value of having real-time monitoring of allowable noise levels during audiometry testing.

Limitations of the current study include the exclusion of control conditions in a sound-treated room, because of the challenging immunocompromised nature of cancer patients. Additionally, no external sound-level measurements apart from the smartphone monitoring included in the mobile health application was employed to monitor environmental noise.

## Conclusion

In conclusion, the current study demonstrated the usefulness of using mobile health audiometry including extended high frequency testing in ototoxicity surveillance for cancer patients receiving platinum-based chemotherapy. Changes in hearing ability over time could be tracked by employing baseline and exit testing. Shortened monitoring protocols focusing on high frequencies and extended high frequencies may be more efficient and address the possibility of noise interference in the lower frequencies during testing. Monitoring can take place at chemotherapy in- and out-patient treatment venues and decrease the patient's already over-burdened treatment schedule.
